# Multiple Epistasis Interactions Within MHC Are Associated With Ulcerative Colitis

**DOI:** 10.3389/fgene.2019.00257

**Published:** 2019-04-03

**Authors:** Jie Zhang, Zhi Wei, Christopher J. Cardinale, Elena S. Gusareva, Kristel Van Steen, Patrick Sleiman, International IBD Genetics Consortium, Hakon Hakonarson

**Affiliations:** ^1^Department of Computer Science, New Jersey Institute of Technology, Newark, NJ, United States; ^2^Adobe Inc., San Jose, CA, United States; ^3^The Children's Hospital of Philadelphia, Center for Applied Genomics, Philadelphia, PA, United States; ^4^GIGA-R Medical Genomics - BIO3, University of Liege, Avenue de l'Hôpital 11, Liège, Belgium; ^5^WELBIO—Walloon Excellence in Life Sciences and BIOtechnology, Liège, Belgium; ^6^Division of Human Genetics, Department of Pediatrics, The Perelman School of Medicine, University of Pennsylvania, Philadelphia, PA, United States

**Keywords:** epistasis, genome-wide association study, immunochip, major histocompatibility complex, ulcerative colitis

## Abstract

Successful searching for epistasis is much challenging, which generally requires very large sample sizes and/or very dense marker information. We exploited the largest Crohn's disease (CD) dataset (18,000 cases + 34,000 controls) and ulcerative colitis (UC) dataset (14,000 cases + 34,000 controls) to date. Leveraging its dense marker information and the large sample size of this IBD dataset, we employed a two-step approach to exhaustively search for epistasis. We detected abundant genome-wide significant (*p* < 1 × 10^−13^) epistatic signals, all within the MHC region. These signals were reduced substantially when conditional on the additive background, but still nine pairs remained significant at the Immunochip-wide level (*P* < 1.1 × 10^−8^) in conditional tests for UC. All these nine epistatic interactions come from the MHC region, and each explains on average 0.15% of the phenotypic variance. Eight of them were replicated in a replication cohort. There are multiple but relatively weak interactions independent of the additive effects within the MHC region for UC. Our promising results warrant the search for epistasis in large data sets with dense markers, exploiting dependencies between markers.

## Introduction

Genome-wide association studies (GWAS) have been conducted widely to interrogate the genetic architecture of common and complex diseases (McCarthy et al., 2008). For Crohn's disease (CD) and ulcerative colitis (UC), the two common forms of inflammatory bowel disease (IBD), GWAS have been fruitful in identifying their susceptibility loci with independent, additive, and cumulative effects (Franke et al., 2010; Anderson et al., 2011; Jostins et al., 2012). Like most other GWAS, these studies have employed a single-locus analysis strategy, namely, testing the variants one at a time for association. Complementary to single-locus analysis, searching for gene-gene interactions, or epistasis, has also attracted extensive research interest in the past decades (Cordell, 2009). However, in contrast to the fruitful achievements of identifying independent additive effects, the success of searching for epistasis is very limited so far.

For IBD, epistasis in CD was once searched in an exhaustive epistatic SNP association analysis on the expanded Wellcome Trust data of seven complex diseases. However, no significant epistasis in CD was identified (Lippert et al., 2013). Indeed, searching for epistasis is much more challenging than detecting additive effects for various reasons, including weaker linkage disequilibrium capturing for a pair of tagging SNPs, increased model complexity, and curse of dimensionality. Very dense marker information and very large sample sizes therefore are required to overcome these challenges (Wei et al., 2014), as well as standardized analysis protocols (Gusareva and Van Steen, 2014).

The Immunochip®, a custom Illumina genotyping microarray, is designed to perform both deep replication of suggestive associations and fine mapping of established GWAS significant loci of major autoimmune and inflammatory diseases (Parkes et al., 2013). For each disease, about 3,000 top-ranked SNPs are selected from available GWAS data. At loci with established disease associations, it includes all known SNPs in the dbSNP database, from the 1000 Genomes project (Feb. 2010 release), and from any other sequencing initiatives that were available to the consortium. As a result, it has in total 196,524 variants, including 718 small insertion deletions and 195,806 SNPs. Thus, it provides a more comprehensive catalog of the most promising candidate variants by picking up the remaining common variants and rare variants that are missed in the first generation of GWAS. Recently, using three large Immunochip datasets, Wei et al. confirmed multiple interactions within the major histocompatibility complex (MHC) and reported novel non-MHC epistatic signals of suggestive significance in their analyses of epistasis in rheumatoid arthritis (Wei et al., 2016).

Here we used the largest data set to date for IBD compiled by the International IBD Genetics Consortium's from its members Immunochip projects to examine epistasis in IBD. Leveraging its dense marker information and the large sample size of this IBD dataset, we searched for epistasis in hope to identify gene-gene interactions for IBD that were missed in previous single-locus analysis.

## Materials and Methods

### Subjects, Genotyping, and Quality Control

We used the large IBD cohort samples from the International IBD Genetics Consortium. These cohort samples have been described in detail elsewhere (Jostins et al., 2012). Briefly, a total of 68,427 samples were recruited from 15 European countries, including 18,227 CD cases with 34,050 CD controls and 14,224 UC cases with 33,954 UC controls, and typed by 11 different genotyping centers on the Immunochip. As shown in Table 1, we randomly split the dataset into a discovery cohort and a replication cohort, each with an approximately equal size (See Tables S1, S2 for details). We refer to the MHC as residing between 28.7 and 34.0 Mb on chromosome 6, based on SNP genomic locations in the GRCh38/hg38 version throughout.

**Table 1:** Cohort information.

**Cohort**	**#CD case**	**#CD CTRL**	**#UC case**	**#UC CTRL**
Discovery cohort	9,125	16,662	7,083	16,578
Replication cohort	9,102	17,388	7,141	17,376
Total	18,227	34,050	14,224	33,954

The IBD dataset used has gone through rigorous quality control (QC) by the IBD consortium (Jostins et al., 2012). Briefly, initial SNP QC was conducted by removing SNPs that fail Hardy-Weinberg equilibrium (HWE) tests across the entire collection or within each batch, SNPs that have significant missing genotypes across the entire collection or within each batch, and SNPs that have different missing genotype rates in case vs. control. The sample QC followed by removing individuals who have a high missing genotype rate, individuals who show significant heterozygosity rate, and duplicated/related individuals. Then, another round of SNP QC was conducted by removing SNPs that show heterogeneous allele frequencies across batches, and SNPs not identified in 1000G project phase 2. For our epistasis analysis, we performed further QC by filtering out markers with Hardy-Weinberg equilibrium *P* < 10^−6^ and minor allele frequency <10^−5^, which results in 149,532 and 150,424 markers for CD and UC, respectively.

### Statistical Analysis

We employed PLINK (Purcell et al., 2007) with default parameters (i.e., “--logistic --hide-covar --adjust”) to perform a GWAS in each cohort using a logistic regression model with 5 principal components and the batch indicators as covariates. The consensus genome-wide significance threshold of 5 × 10^−8^ was applied. We expect that many of these genome-wide significant SNPs are correlated. To obtain independent signals, we further pooled the selected SNPs (*P* < 5 × 10^−8^) and re-fit the logistic regression model using all of them. For the fully correlated SNPs, only one will be kept. Then we considered the SNPs with *P* < 0.05 from the logistic regression fitted with all marginally genome-wide significant SNPs, as independent additive signals. As we describe later, these significant independent GWAS SNPs will be used as the additive background to screen the SNP pairs we identify, for ensuring they are truly novel epistasis signals that are not captured by any single additive signals.

We used a 2-step approach to detect epistasis. First, we utilized the fast approximate tests provided by BOOST (Wan et al., 2010) to screen candidate gene-gene interactions. BOOST can perform quickly a full pairwise screening without correction for covariates in the discovery cohort. The BOOST *P*-values were approximate and we retained all possible epistatic pairs of SNPs with an interaction *P* < 10^−10^ and *r*^2^ < 0.2. We subsequently computed accurate *P*-values for the retained SNP pairs using a full logistic regression model accounting for population stratification and batch effect covariates. The epistatic interaction was tested using a likelihood ratio test with 4 degrees of freedom as previously described (Gyenesei et al., 2012). Following Wei et al. (2016), we adopted two *P*-value thresholds, 10^−13^ for claiming genome-wide significant epistatic SNP pairs (Wei et al., 2014), and 1.1 × 10^−8^ for claiming Immunochip-wide 5% significance.

Finally, to identify significant epistatic SNP pairs conditioning on the additive background, we added the selected independent GWAS SNPs as covariates into the logistic regression model for testing the epistasis. Variance explained by the selected epistatic SNP pairs was estimated using a full logistic regression model including all the covariates, independent SNPs and SNP pairs. The SNP pairs identified in the discovery cohort were tested similarly in the replication cohort. We considered a pair to be directly replicated if its epistatic *P*-value remained <0.05 after conditioning on the additive background.

## Results

The univariate GWAS scan of the discovery cohorts identified 2,765 and 1,123 genome-wide significant SNPs (*P* < 5 × 10^−8^) for CD and UC, respectively. After the further independence screening, we obtained 306 and 121 independent SNPs for CD and UC, respectively. The detailed information about the SNPs selected in each stage were presented in the Supplemental Data. The full pairwise scan by BOOST produced 13,843 and 35,373 candidate pairs of SNPs that have BOOST interaction *P* < 10^−10^ and *r*^2^ < 0.2 for CD and UC, respectively. We computed their accurate *P*-values, and detected 11 and 513 genome-wide significant pairs (*P* < 10^−13^) for CD and UC, respectively. Conditioning on the additive background of the 306 independent CD SNPs, none of the 11 CD pairs remained significant (smallest *P* = 3.0 × 10^−4^). For UC, we obtained 9 pairs significant at the Immunochip-wide level (*P* < 1.1 × 10^−8^), and all of them came from the MHC region. Conditional on the additive background, these epistatic pairs jointly explain an additional 0.49% of the phenotypic variance on the observed scale, of which 0.36% by interactions only, suggesting that these interaction effects were not negligible jointly, but weak individually (i.e., on average explained 0.15% of the phenotypic variance). Except for the pair of lowest significance, the top 8 of these 9 pairs were replicated in the independent replication cohort with *P* < 0.05 (See Table 2 and Figure S1 for details).

**Table 2:** Independent epistatic SNP pairs within the MHC region adjusted for the pre-identified additive background in the discovery cohort and statistically replicated in an independent replication cohort.

							**Discovery Cohort**				**Replication cohort**			
**SNP1**	**Pos1**	**Gene1**	**SNP2**	**Pos2**	**Gene2**	**LD**	**P_**SNP1**_**	**P_**SNP2**_**	**P_**SNP1xSNP2**_**	**P_**SNP1xSNP2**_(Cond)**	**P_**SNP1**_**	**P_**SNP2**_**	**P_**SNP1xSNP2**_**	**P_**SNP1xSNP2**_(Cond)**
rs532098	32610275		rs6928482	32658472	~HLA-DQB1-AS1	0.197	2.22E-33	2.88E-10	7.83E-21	3.12E-21	9.74E-30	2.30E-10	2.16E-08	4.79E-03
rs13207945	32611931		rs6928482	32658472	~HLA-DQB1-AS1	0.196	1.74E-33	2.88E-10	1.17E-20	3.56E-21	7.98E-30	2.30E-10	2.11E-08	4.61E-03
rs482044	32608287		rs6928482	32658472	~HLA-DQB1-AS1	0.162	2.21E-22	2.88E-10	3.60E-21	4.28E-16	2.57E-21	2.30E-10	4.34E-09	5.39E-03
rs9272143	32633026		rs6928482	32658472	~HLA-DQB1-AS1	0.103	3.10E-13	2.88E-10	3.14E-43	2.14E-12	2.29E-08	2.30E-10	2.25E-29	3.15E-03
rs3852215	32667724	~HLA-DQB1	rs7744001	32658309	~HLA-DQB1-AS1	0.021	2.33E-08	7.26E-08	7.50E-14	3.11E-11	3.71E-11	7.80E-04	1.11E-04	4.07E-05
rs7774434	32689801		rs6928482	32658472	~HLA-DQB1-AS1	0.023	8.43E-06	2.88E-10	3.30E-22	1.21E-10	8.12E-07	2.30E-10	4.22E-13	8.51E-03
rs9272143	32633026		rs7744001	32658309	~HLA-DQB1-AS1	0.003	3.10E-13	7.26E-08	1.27E-16	2.07E-10	2.29E-08	7.80E-04	6.65E-10	1.96E-03
rs9272105	32632222		rs6928482	32658472	~HLA-DQB1-AS1	0.067	7.53E-14	2.88E-10	1.10E-39	6.76E-10	9.78E-08	2.30E-10	8.96E-26	2.36E-02
rs1964995	32481634		rs6928482	32658472	~HLA-DQB1-AS1	0.133	9.64E-25	2.88E-10	2.76E-23	3.32E-09	2.33E-12	2.30E-10	1.26E-09	1.05E-01

**Figure 1:**
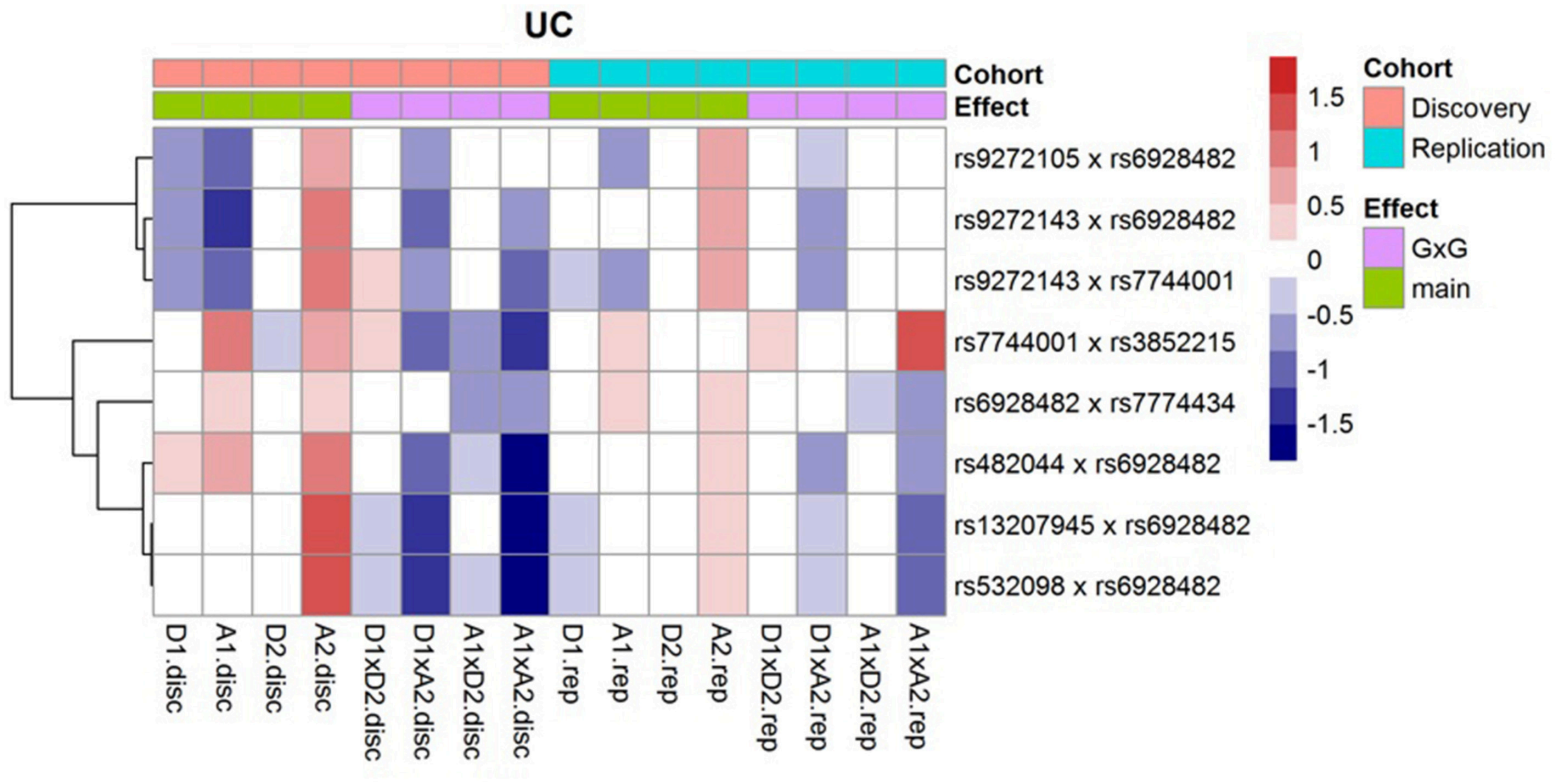
Effects of the orthogonal additive (A1, A2), dominant (D1, D2), and epistatic effects (A1xA2, A1xD2, D1xA2, D1xD2) for the 8 replicated UC interactions.

Following Hemani et al. (2014), we decompose the genetic effects of each of the SNP pairs into orthogonal additive (A1, A2), dominant (D1, D2) and epistatic effects (A1xA2, A1xD2, D1xA2, D1xD2); and then display them (regression coefficients) as a heatmap (Figure 1). For these interactions, we observe that the epistatic effects (A1xA2, A1xD2, D1xA2, D1xD2) generally act in opposite direction against the main effects (A1, A2, D1, D2). In addition, we note that the effects across the discovery and replication cohorts are largely concordant.

All the 10 SNPs contributing to the 8 epistatic pairs are non-coding, with rs3852215 close to HLA-DQB1, and rs6928482 and rs7744001 close to HLA-DQB1-AS1. It has been known for some time that the single strongest genetic association for IBD is the HLA-DRB1^*^103 allele, which is located within the MHC region (Silverberg et al., 2003). A recent study by Goyette et al. demonstrated the importance of HLA-DRB1^*^0103 in both CD and UC by genotyping 7,406 MHC SNPs in 32,000 IBD cases and an equal number of controls (Goyette et al., 2015) The fine resolution of mapping allowed localization of the association signal to specific amino acid substitutions in the MHC molecule which revealed that the causal variants are located within the peptide binding groove and thereby influence antigen presentation directly (Goyette et al., 2015). The mechanism by which these mutations produce autoimmunity could be by enabling self- or commensal-antigenic peptides to bind and be presented to helper T cells. The non-coding SNPs identified in our study which have gene-gene interaction with these high-risk variants could affect transcriptional regulation of the high-risk MHC molecules themselves, enabling greater amounts of self or commensal antigens to be presented in a differential manner provoking an inflammatory response.

## Discussion

In this study we present results from an epistatic analysis of a large data set from the International IBD Genetics Consortium genotyped on the Immunochip array. Most previous studies have used a relatively small sample sizes (with <2,000 cases and 3,000 controls) and a GWAS array (Wan et al., 2010; Lippert et al., 2013), while here we had the largest IBD dataset to date with a much increased sample size (14,000+ cases and 30,000+ controls) genotyped on the high-density Immunochip. The large sample size has enabled us to identify genome-wide significant interactions within the MHC region for UC for the first time. All the 8 replicated epistasis signals are local interactions (within a distance <1 Mb), which is consistent with recent finding that examining local interactions between SNP closely located but with low LD (*r*^2^ < 0.2) could increase the power of detection of missing variants and/or functional interactions (Wei et al., 2013, 2014). It is noted that 3 of these 8 interaction signals are genome-wide significant and the other 5 remain Immunochip-wide significant after conditioning on the additive background. These independent interactions each with a substantial effect were statistically replicated in an independent replication cohort. These results confirm the increased level of complexity in the entire MHC region as observed also in rheumatoid arthritis (Wei et al., 2016), namely, there can still exist additional epistatic interactions over and above the well-established multiple independent MHC signals. The current resolution provided by the Immunochip SNP resolution should be able to identify most MHC diversity. Even if some additive background may be derived imperfectly, the large sample size of the IBD dataset should compensate and lend sufficient power for capturing all the additive background comprehensively. Therefore, the 8 epistatic pairs we report here should be independent from the additive background.

Finally, we would like to point out that multiple views to genome-wide data for epistasis screening exist. In this work, we developed a protocol that lead to replicable statistical results. Notably, small changes in the protocol (including the use of prior biological knowledge about the disease, LD pruning, analytic methodology, correction for population structure or multiple testing) may give rise to widely varying results (Bessonov et al., 2015). More work is needed to investigate the relation between MHC susceptibility genes and their relation with other genomic regions, aiding the hunt for non-MHC driven epistasis.

In conclusion, by leveraging the large sample size available through the International IBD Genetics Consortium genotyped on the Immunochip, we have identified and replicated concordant epistatic interactions within the MHC region for UC. Further examination of these identified epistatic interactions may help to understand the molecular mechanisms underlying epistatic interactions with the MHC locus and their contributions to immunological diseases. Our promising results warrant the search for epistasis in large data sets to address the missing heritability in complex disease. Optimal epistasis analysis protocols need to be derived in order to exploit the richness potentially harbored by dense marker panels.

## Data Availability

Data have been deposited in NCBI's database of Genotypes and Phenotypes (dbGaP) through study accession numbers phs000130.v1.p1 and phs000345.v1.p1.

## Author Contributions

ZW and HH conceptualized and led the study. JZ and ZW performed the experiments and analyses. JZ, ZW, CC, ESG, KVS, and HH contributed to writing. PS and HH contributed to samples and phenotypes.

### Conflict of Interest Statement

JZ is employed by company Adobe. The remaining authors declare that the research was conducted in the absence of any commercial or financial relationships that could be construed as a potential conflict of interest. The reviewer XY declared a shared affiliation, with no collaboration, with one of the authors, PS, to the handling editor at time of review.
