# Supplementary material for: Multiple Epistasis Interactions Within MHC Are Associated With Ulcerative Colitis
Source: Front Genet. 2019 Apr 3;10:257. doi: 10.3389/fgene.2019.00257 (PMC6456704; doi:10.3389/fgene.2019.00257)
Supplement: Supplementary file 1 [file Presentation_1.pdf]

Multiple Epistasis Interactions within MHC are associated with Ulcerative Colitis

Jie Zhang PhD[1,2], Zhi Wei PhD[1,*], Christopher J Cardinale MD, PhD[3], Elena S. Gusareva PhD[4], Kristel Van Steen PhD[4,5], Patrick Sleiman PhD[3,6], International IBD Genetics Consortium, and Hakon Hakonarson MD, Ph[3,6,*]

1. Department of Computer Science, New Jersey Institute of Technology, Newark, NJ
2. Adobe Inc., San Jose, CA
3. Center for Applied Genomics, The Children's Hospital of Philadelphia, Philadelphia, PA
4. GIGA-R Medical Genomics - BIO3, University of Liege, Avenue de l'Hôpital 11, 4000 Liege, Belgium
5. WELBIO—Walloon Excellence in Life Sciences and BIOtechnology, Belgium
6. Division of Human Genetics, Department of Pediatrics, The Perelman School of Medicine, University of Pennsylvania, Philadelphia, PA

*** Correspondence:**

Zhi Wei Ph.D.: zhiwei@njit.edu
Department of Computer Science
New Jersey Institute of Technology
Newark, NJ 07102

Hakon Hakonarson M.D., Ph.D: hakonarson@email.chop.edu
Center for Applied Genomics
The Children's Hospital of Philadelphia
Philadelphia, PA 19104

Supplementary Materials for:

Multiple Epistasis Interactions within MHC are associated with Crohn's Disease

Table S1: Discovery Cohorts

BATCH	#CD CASE	#CD CTRL	#UC CASE	#UC CTRL
dellinghaus_icbatch1	2,419	3,266	795	3,266
dellinghaus_icbatch2	0	168	100	168
dellinghaus_icbatch3	276	141	109	140
dellinghaus_icbatch4	0	913	0	913
IMSGC	0	5,740	0	5,740
kfransen_icbatch1	1,152	727	293	727
kfransen_icbatch2	82	871	137	871
ljostins_icbatch1	1,239	83	1,044	0
ljostins_icbatch2	0	1,271	0	1,271
niddk_icbatch1	2,778	1,345	2,313	1,345
niddk_icbatch2	834	399	673	399
niddk_icbatch3	24	42	24	42
rduerr_icbatch1	0	1,357	1,407	1,357
rduerr_icbatch2	321	322	188	322
rduerr_icbatch3	0	17	0	17
Total	9,125	16,662	7,083	16,578

Table S2: Replication Cohorts

BATCH	#CD CASE	#CD CTRL	#UC CASE	#UC CTRL
etheatre_icbatch1	780	636	479	636
etheatre_icbatch2	329	77	80	77
jbarrett_icbatch1	2,377	4,291	2,711	4,290
jbarrett_icbatch2	276	0	104	1
pgregersen_icbatch2	0	1,611	0	1,611
srich_icbatch1	0	4,259	0	4,259
ssommeren_icbatch2	77	107	201	107
svermeire_icbatch1	433	484	466	484
svermeire_icbatch2	1,063	298	321	298
svermeire_icbatch3	38	120	45	120
tbalschun_icbatch1	1,839	1,654	1,683	1,642
tbalschun_icbatch2	0	1,496	0	1,496
tbalschun_icbatch3	0	2,037	0	2,037
tbalschun_icbatch4	0	318	0	318
tharitunians_icbatch1	1,326	0	823	0
tharitunians_icbatch2	330	0	53	0
tharitunians_icbatch3	234	0	175	0
Total	9,102	17,388	7,141	17,376

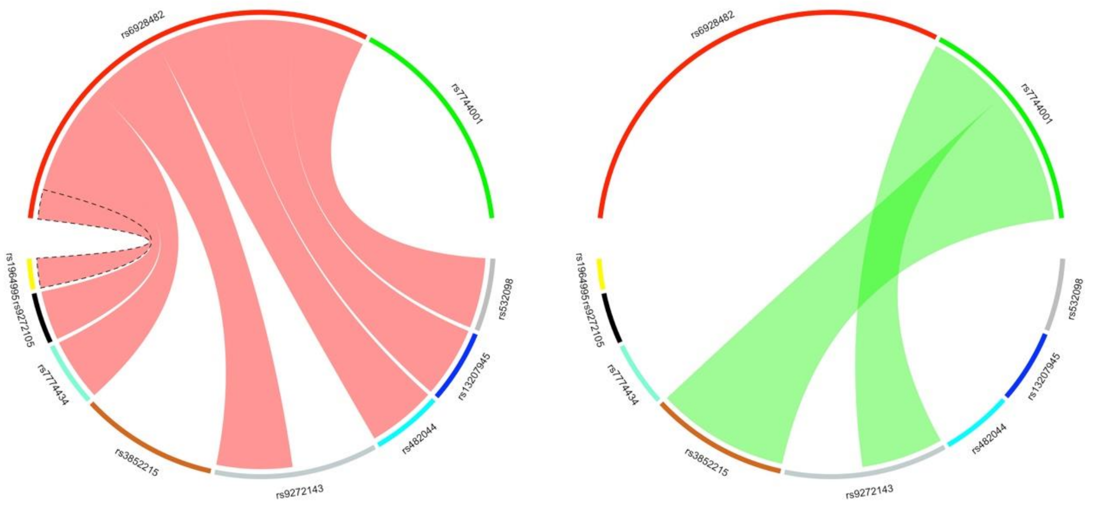

Figure S1: Circular visualization for the independent epistatic SNP pairs shown in Table 2. The link width represents the significance level and all SNP pairs (except the pair rs1964995 and rs6928482 denoted by link with dotted line) could be replicated in the independent replication cohort with $P < 0.05$.

International IBDGC Contributing Members:

Murray Barclay[1], Laurent Peyrin-Biroulet[2], Mathias Chamaillard[3], Jean-Frederick Colombel[4], Mario Cottone[5], Anthony Croft[6], Renata D'Incà[7], Jonas Halfvarson[8,9], Katherine Hanigan[6], Paul Henderson[10,11], Jean-Pierre Hugot[12,13], Amir Karban[14], Nicholas A Kennedy[15], Mohammed Azam Khan[16], Marc Lémann[17], Arie Levine[18], Dunecan Massey[19], Monica Milla[20], Grant W Montgomery[21], Sok Meng Evelyn Ng[22], Ioannis Oikonomou[22], Harald Peeters[23], Deborah D. Proctor[22], Jean-Francois Rahier[24], Rebecca Roberts[2], Paul Rutgeerts[25], Frank Seibold[26], Laura Stronati[27], Kirstin M Taylor[28], Leif Törkvist[29], Kullak Ublick[30], Johan Van Limbergen[31], Andre Van Gossum[32], Morten H. Vatn[33], Hu Zhang[20], Wei Zhang[22], Australia and New Zealand IBDGC*, Belgium Genetic Consortium†, Initiative on Crohn and Colitis, NIDDK IBDGC‡, United Kingdom IBDGC, Wellcome Trust Case Control Consortium§

[1]Department of Medicine, University of Otago, Christchurch, New Zealand. [2]Gastroenterology Unit, INSERM U954, Nancy University and Hospital, France. [3]INSERM, U1019, Lille, France. [4]Univ Lille Nord de France, CHU Lille and Lille-2 University,Gastroenterology Unit, France. [5]Division of Internal Medicine, Villa Sofia-V. Cervello Hospital, University of Palermo, Palermo, Italy. [6]Inflammatory Bowel Diseases, Genetic Epidemiology, Queensland Institute of Medical Research, Brisbane, Australia. [7]Department of Surgical and Gastroenterological Sciences, University of Padua, Padua, Italy. [8]Department of Medicine, Örebro University Hospital, Örebro, Sweden. [9]School of Health and Medical Sciences, Örebro University, Örebro, Sweden. [10]Royal Hospital for Sick Children, Paediatric Gastroenterology and Nutrition, Edinburgh, UK. [11]Child Life and Health, University of Edinburgh, Edinburgh, UK. [12]INSERM U843, Paris, France. [13]Univ-Paris Diderot Sorbonne Paris-Cité, Paris France. [14]Department of Gastroenterology, Faculty of Medicine, Technion-Israel Institute of Technology, Haifa, Israel. [15]Gastrointestinal Unit, Institute of Genetics and Molecular Medicine, University of Edinburgh, Edinburgh, UK. [16]Genetic Medicine, MAHSC, University of Manchester, Manchester, UK. [17]Université Paris Diderot, GETAID group, Paris, France. [18]Pediatric Gastroenterology Unit, Wolfson Medical Center and Sackler School of Medicine, Tel Aviv University, Tel Aviv, Israel. [19]Inflammatory Bowel Disease Research Group, Addenbrooke's Hospital, University of Cambridge, Cambridge, UK. [20]Azienda Ospedaliero Universitaria (AOU) Careggi, Unit of Gastroenterology SOD2, Florence, Italy. [21] Molecular Epidemiology, Queensland Institute of Medical Research, Brisbane, Australia. [22]Department of Internal Medicine, Section of Digestive Diseases, Yale School of Medicine, New Haven, Connecticut, USA. [23]Dept Gastroenterology - University hospital Gent - De Pintelaan - 9000 Gent Belgium. [24]Dept Gastroenterology - UCL Mont Godinne Belgium. [25]Division of Gastroenterology, University Hospital Gasthuisberg, Leuven, Belgium. [26]University of Bern, Division of Gastroenterology, Inselspital, Bern, Switzerland. [27]Department of Radiobiology and Human Health, Italian National Agency for New Technologies, Energy and Sustainable Economic Development (ENEA), Rome, Italy. [28]Dept Gastroenterology, Guy's & St Thomas' NHS Foundation Trust, St Thomas' Hospital, London, UK. [29]Department of Clinical Science, Intervention and Technology, Karolinska Institutet,

Stockholm, Sweden. [30]Division of Clinical Pharmacology and Toxicology, University Hospital Zurich, Zurich, Switzerland. [31]Division of Pediatric Gastroenterology, Hepatology and Nutrition, Hospital for Sick Children, Toronto, Ontario, Canada. [32]Dept Gastroenterology - 3University Brussels. [33] Department of Transplantation Medicine, Division of Cancer medicine, Surgery and Transplantation, Oslo University Hospital Rikshospitalet, Oslo, Norway.

***Australia and New Zealand IBDGC**

Jane M. Andrews[1], Peter A. Bampton[2], Murray Barclay[3], Timothy H. Florin[4], Richard Gearry[3], Krupa Krishnaprasad[5], Ian C. Lawrance[6], Gillian Mahy[7], Grant W. Montgomery[8], Graham Radford-Smith[5,9], Rebecca L. Roberts[10], Lisa A. Simms[5].

[1]Inflammatory Bowel Disease Service, Department of Gastroenterology and Hepatology, Royal Adelaide Hospital, and School of Medicine, University of Adelaide, Adelaide, Australia. [2]Department of Gastroenterology and Hepatology, Flinders Medical Centre and School of Medicine, Flinders University, Adelaide, Australia. [3]Department of Gastroenterology, Christchurch Hospital and Department of Medicine, University of Otago, Christchurch, New Zealand. [4]Department of Gastroenterology, Mater Health Services, Brisbane, Australia, and School of Medicine, University of Queensland, Brisbane, Australia. [5]Inflammatory Bowel Diseases, Genetics and Computational Biology, Queensland Institute of Medical Research, Brisbane, Australia. [6]Centre for Inflammatory Bowel Diseases, Fremantle Hospital and School of Medicine and Pharmacology, The University of Western Australia, Fremantle, Australia. [7]Department of Gastroenterology, The Townsville Hospital and James Cook University School of Medicine, Townsville, Australia. [8]Molecular Epidemiology, Genetics and Computational Biology, Queensland Institute of Medical Research, Brisbane, Australia. [9]Department of Gastroenterology, Royal Brisbane and Womens Hospital, and School of Medicine, University of Queensland, Brisbane, Australia. [10]University of Otago, Department of Medicine, Christchurch, New Zealand.

†Belgium Genetic Consortium

Leila Amininijad[1], Isabelle Cleynen[2], Olivier Dewit[3], Denis Franchimont[1], Michel Georges[4], Debby Laukens[5], Harald Peeters[5], Jean-Francois Rahier[3], Paul Rutgeerts[2], Emilie Theatre[4], [6], André Van Gossum[1], Severine Vermeire[7].

[1]Erasmus Hospital, Free University of Brussels, Department of Gastroenterology, Brussels, Belgium. [2]Department of Pathophysiology, Gastroenterology section, KU Leuven, Leuven, Belgium. [3]Department of Gastroenterology, Clinique Universitaire St-Luc, Brussels, Belgium. [4]Unit of Animal Genomics, Groupe Interdisciplinaire de Gnoprotomique Applique (GIGA-R) and Faculty of Veterinary Medicine, University of Lige, Lige, Belgium. [5]Ghent University Hospital, Department of Gastroenterology and Hepatology, Ghent, Belgium. [6]Division of Gastroenterology, Centre Hospitalier

Universitaire, Universit de Lige, Lige, Belgium. [7]Division of Gastroenterology, University Hospital Gasthuisberg, Leuven, Belgium.

‡NIDDK Inflammatory Bowel Disease Genetics Consortium‡

Guy Aumais[1], Leonard Baidoo[2], Arthur M. Barrie III[2], Karen Beck[2], Edmond-Jean Bernard[3], David G. Binion[2], Alain Bitton[4], Steve R. Brant[5], Judy H. Cho[6,7], Albert Cohen[8], Kenneth Croitoru[9], Mark J. Daly[10,11], Lisa W. Datta[5], Colette Deslandres[12], Richard H. Duerr[2,13], Debra Dutridge[14], John Ferguson[7], Joann Fultz[2], Philippe Goyette[15], Gordon R. Greenberg[9], Talin Haritunians[14], Gilles Jobin[16], Seymour Katz[17], Raymond G. Lahaie[18], Dermot P. McGovern[14,19], Linda Nelson[2], Sok Meng Ng[7], Kaida Ning[7], Ioannis Oikonomou[7], Pierre Paré[20], Deborah D. Proctor[7], Miguel D. Regueiro[2], John D. Rioux[15], Elizabeth Ruggiero[7], L. Philip Schumm[21], Marc Schwartz[2], Regan Scott[2], Yashoda Sharma[7], Mark S. Silverberg[9], Denise Spears[5], A. Hillary Steinhart[9], Joanne M. Stempak[9], Jason M. Swoger[2], Constantina Tsagarelis[4], Wei Zhang[7], Clarence Zhang[22], Hongyu Zhao[22].

[1]University of Montreal, Maissonneuve – Rosemont Hospital, Quebec Association of Gastroenterologists, Montréal, Québec, Canada. [2]Division of Gastroenterology, Hepatology and Nutrition, Department of Medicine, University of Pittsburgh School of Medicine, Pittsburgh, Pennsylvania, USA. [3]Hôpital Hôtel Dieu, Montréal, Québec, Canada. [4]Division of Gastroenterology, McGill University Health Centre, Royal Victoria Hospital, Montréal, Québec, Canada. [5]Inflammatory Bowel Disease Center, Department of Medicine, Johns Hopkins University School of Medicine, Baltimore, Maryland, USA. [6]Department of Genetics, Yale School of Medicine, New Haven, Connecticut, USA. [7]Department of Internal Medicine, Section of Digestive Diseases, Yale School of Medicine, New Haven, Connecticut, USA. [8]Division of Gastroenterology, Hôpital Général Juif Sir Mortimer B. Davis Jewish General Hospital, Montréal, Québec, Canada. [9]Mount Sinai Hospital Inflammatory Bowel Disease Centre, University of Toronto, Toronto, Ontario, Canada. [10]Analytic and Translational Genetics Unit, Massachusetts General Hospital, Harvard Medical School, Boston, Massachusetts, USA. [11]Broad Institute of MIT and Harvard, Cambridge, Massachusetts, USA. [12]Hopital Sainte Justine, Montréal, Québec, Canada. [13]Department of Human Genetics, University of Pittsburgh Graduate School of Public Health, Pittsburgh, Pennsylvania, USA. [14]Medical Genetics Institute, Cedars-Sinai Medical Center, Los Angeles, California, USA. [15]Université de Montréal and the Montreal Heart Institute, Research Center, Montréal, Québec, Canada. [16]Pavillon Maisonneuve, Montréal, Québec, Canada. [17]Long Island Clinical Research Associates, Great Neck, New York, USA. [18]CHUM – Hopital Sainte-Luc, Montréal, Québec, Canada. [19]Inflammatory Bowel and Immunobiology Research Institute, Cedars-Sinai Medical Center, Los Angeles, California, USA. [20]Laval University, Quebec City, Québec, Canada. [21]Department of Health Studies, University of Chicago, Chicago, Illinois, USA. [22]Department of Biostatistics, School of Public Health, Yale University, New Haven, Connecticut, USA.

§Members of the Wellcome Trust Case Control Consortium

Jan Aerts[1], Tariq Ahmad[2], Hazel Arbury[1], Anthony Attwood[1,3,4], Adam Auton[5], Stephen G Ball[6], Anthony J Balmforth[6], Chris Barnes[1], Jeffrey C Barrett[1], Inês Barroso[1], Anne Barton[7], Amanda J Bennett[8], Sanjeev Bhaskar[1], Katarzyna Blaszczyk[9], John Bowes[7], Oliver J Brand[8,10], Peter S Braund[11], Francesca Bredin[12], Gerome Breen[13,14], Morris J Brown[15], Ian N Bruce[7], Jaswinder Bull[16], Oliver S Burren[17], John Burton[1], Jake Byrnes[18], Sian Caesar[19], Niall Cardin[5], Chris M Clee[1], Alison J Coffey[1], John MC Connell[20], Donald F Conrad[1], Jason D Cooper[17], Anna F Dominiczak[20], Kate Downes[17], Hazel E Drummond[21], Darshna Dudakia[16], Andrew Dunham[1], Bernadette Ebbs[16], Diana Eccles[22], Sarah Edkins[1], Cathryn Edwards[23], Anna Elliot[16], Paul Emery[24], David M Evans[25], Gareth Evans[26], Steve Eyre[7], Anne Farmer[14], I Nicol Ferrier[27], Edward Flynn[7], Alistair Forbes[28], Liz Forty[29], Jayne A Franklyn[10,30], Timothy M Frayling[2], Rachel M Freathy[2], Eleni Giannoulatou[5], Polly Gibbs[16], Paul Gilbert[7], Katherine Gordon-Smith[19,29], Emma Gray[1], Elaine Green[29], Chris J Groves[8], Detelina Grozeva[29], Rhian Gwilliam[1], Anita Hall[16], Naomi Hammond[1], Matt Hardy[17], Pile Harrison[31], Neelam Hassanali[8], Husam Hebaishi[1], Sarah Hines[16], Anne Hinks[7], Graham A Hitman[32], Lynne Hocking[33], Chris Holmes[5], Eleanor Howard[1], Philip Howard[34], Joanna MM Howson[17], Debbie Hughes[16], Sarah Hunt[1], John D Isaacs[35], Mahim Jain[18], Derek P Jewell[36], Toby Johnson[34], Jennifer D Jolley[3,4], Ian R Jones[29], Lisa A Jones[19], George Kirov[29], Cordelia F Langford[1], Hana Lango-Allen[2], G Mark Lathrop[37], James Lee[12], Kate L Lee[34], Charlie Lees[21], Kevin Lewis[1], Cecilia M Lindgren[8,18], Meeta Maisuria-Armer[17], Julian Maller[18], John Mansfield[38], Jonathan L Marchini[5], Paul Martin[7], Dunecan CO Massey[12], Wendy L McArdle[39], Peter McGuffin[14], Kirsten E McLay[1], Gil McVean[5,18], Alex Mentzer[40], Michael L Mimmack[1], Ann E Morgan[41], Andrew P Morris[18], Craig Mowat[42], Patricia B Munroe[34], Simon Myers[18], William Newman[26], Elaine R Nimmo[21], Michael C O'Donovan[29], Abiodun Onipinla[34], Nigel R Ovington[17], Michael J Owen[29], Kimmo Palin[1], Aarno Palotie[1], Kirstie Parnell[2], Richard Pearson[8], David Pernet[16], John RB Perry[2,18], Anne Phillips[42], Vincent Plagnol[17], Natalie J Prescott[9], Inga Prokopenko[8,18], Michael A Quail[1], Suzanne Rafelt[11], Nigel W Rayner[8,18], David M Reid[33], Anthony Renwick[16], Susan M Ring[39], Neil Robertson[8,18], Samuel Robson[1], Ellie Russell[29], David St Clair[13], Jennifer G Sambrook[3,4], Jeremy D Sanderson[40], Stephen J Sawcer[43], Helen Schuilenburg[17], Carol E Scott[1], Richard Scott[16], Sheila Seal[16], Sue Shaw-Hawkins[34], Beverley M Shields[2], Matthew J Simmonds[8,10], Debbie J Smyth[17], Elilan Somaskantharajah[1], Katarina Spanova[16], Sophia Steer[44], Jonathan Stephens[3,4], Helen E Stevens[17], Kathy Stirrups[1], Millicent A Stone[45,46], David P Strachan[47], Zhan Su[5], Deborah PM Symmons[7], John R Thompson[48], Wendy Thomson[7], Martin D Tobin[48], Mary E Travers[8], Clare Turnbull[16], Damjan Vukcevic[18], Louise V Wain[48], Mark Walker[49], Neil M Walker[17], Chris Wallace[17], Margaret Warren-Perry[16], Nicholas A Watkins[3,4], John Webster[50], Michael N Weedon[2], Anthony G Wilson[51], Matthew Woodburn[17], B Paul Wordsworth[52], Chris Yau[5], Allan H Young[27,53], Eleftheria Zeggini[1], Matthew A Brown[52,54], Paul R Burton[48], Mark J Caulfield[34], Alastair Compston[43], Martin Farrall[55], Stephen CL Gough[8,10,30], Alistair S Hall[6], Andrew T Hattersley[2,56], Adrian VS Hill[18], Christopher G Mathew[9], Marcus Pembrey[57], Jack Satsangi[21], Michael R Stratton[1,16], Jane Worthington[7], Matthew E Hurles[1], Audrey Duncanson[58], Willem H Ouwehand[1,3,4], Miles Parkes[12], Nazneen Rahman[16], John A Todd[17], Nilesh J Samani[11,59], Dominic P Kwiatkowski[1,18], Mark I McCarthy[8,18,60], Nick Craddock[29], Panos Deloukas[1], Peter Donnelly[5,18], Jenefer M Blackwell[61, 62], Elvira Bramon[63], Juan P Casas[64] , Aiden Corvin[65], Janusz Jankowski[66], Hugh S

Markus[67], Colin NA Palmer[68], Robert Plomin[14], Anna Rautanen[18], Richard C Trembath[9], Ananth C Viswanathan[69], Nicholas W Wood[70], Chris C A Spencer[18], Gavin Band[18], Céline Bellenguez[18], Colin Freeman[18], Garrett Hellenthal[18], Eleni Giannoulatou[18], Matti Pirinen[18], Richard Pearson[18], Amy Strange[18], Hannah Blackburn[1], Suzannah J Bumpstead[1], Serge Dronov[1], Matthew Gillman[1], Alagurevathi Jayakumar[1], Owen T McCann[1], Jennifer Liddle[1], Simon C Potter[1], Radhi Ravindrarajah[1], Michelle Ricketts[1], Matthew Waller[1], Paul Weston[1], Sara Widaa[1], Pamela Whittaker[1].

[1]The Wellcome Trust Sanger Institute, Wellcome Trust Genome Campus, Hinxton, Cambridge, CB10 1SA UK. [2]Genetics of Complex Traits, Peninsula College of Medicine and Dentistry University of Exeter, EX1 2LU, UK.[3]Department of Haematology, University of Cambridge, Long Road, Cambridge, CB2 0PT, UK. [4]National Health Service Blood and Transplant, Cambridge Centre, Long Road, Cambridge CB2 0PT, UK. [5]Department of Statistics, University of Oxford, 1 South Parks Road, Oxford, OX1 3TG, UK. [6]Multidisciplinary Cardiovascular Research Centre (MCRC), Leeds Institute of Genetics, Health and Therapeutics (LIGHT), University of Leeds, Leeds, LS2 9JT, UK. [7]ARC Epidemiology Unit, Stopford Building, University of Manchester, Oxford Road, Manchester, M13 9PT, UK. [8] Oxford Centre for Diabetes, Endocrinology and Medicine, University of Oxford, Churchill Hospital, Oxford OX3 7LJ, UK. [9]Department of Medical and Molecular Genetics, King's College London School of Medicine, 8th Floor Guy's Tower, Guy's Hospital, London, SE1 9RT, UK. [10]Centre for Endocrinology, Diabetes and Metabolism, Institute of Biomedical Research, University of Birmingham, Birmingham, B15 2TT, UK. [11]Department of Cardiovascular Sciences, University of Leicester, Glenfield Hospital, Groby Road, Leicester LE3 9QP, UK. [12] IBD Genetics Research Group, Addenbrooke's Hospital, Cambridge, CB2 0QQ, UK. [13]University of Aberdeen, Institute of Medical Sciences, Foresterhill, Aberdeen AB25 2ZD, UK. [14]SGDP, The Institute of Psychiatry, King's College London, De Crespigny Park, Denmark Hill, London SE5 8AF, UK. [15]Clinical Pharmacology Unit, University of Cambridge, Addenbrookes Hospital, Hills Road, Cambridge CB2 2QQ, UK. [16]Section of Cancer Genetics, Institute of Cancer Research, 15 Cotswold Road, Sutton SM2 5NG, UK. [17]Juvenile Diabetes Research Foundation/Wellcome Trust Diabetes and Inflammation Laboratory, Department of Medical Genetics, Cambridge Institute for Medical Research, University of Cambridge, Wellcome Trust/MRC Building, Cambridge CB2 0XY, UK. [18]The Wellcome Trust Centre for Human Genetics, University of Oxford, Roosevelt Drive, Oxford OX3 7BN, UK. [19]Department of Psychiatry, University of Birmingham, National Centre for Mental Health, 25 Vincent Drive, Birmingham, B15 2FG, UK.[20]BHF Glasgow Cardiovascular Research Centre, University of Glasgow, 126 University Place, Glasgow, G12 8TA, UK. [21]Gastrointestinal Unit, Division of Medical Sciences, School of Molecular and Clinical Medicine, University of Edinburgh, Western General Hospital, Edinburgh EH4 2XU, UK. [22]Academic Unit of Genetic Medicine, University of Southampton, Southampton, UK. [23]Endoscopy Regional Training Unit, Torbay Hospital, Torbay TQ2 7AA, UK. [24]Academic Unit of Musculoskeletal Disease, University of Leeds, Chapel Allerton Hospital, Leeds, West Yorkshire LS7 4SA, UK. [25]MRC Centre for Causal Analyses in Translational Epidemiology, Department of Social Medicine, University of Bristol, Bristol, BS8 2BN, UK. [26]Department of Medical Genetics, Manchester Academic Health Science Centre (MAHSC), University of Manchester, Manchester M13 0JH, UK. [27]School of Neurology, Neurobiology and Psychiatry,

Royal Victoria Infirmary, Queen Victoria Road, Newcastle upon Tyne, NE1 4LP, UK. [28]Institute for Digestive Diseases, University College London Hospitals Trust, London NW1 2BU, UK. [29]MRC Centre for Neuropsychiatric Genetics and Genomics, School of Medicine, Cardiff University, Heath Park, Cardiff, CF14 4XN, UK. [30]University Hospital Birmingham NHS Foundation Trust, Birmingham, B15 2TT, UK. [31]University of Oxford, Institute of Musculoskeletal Sciences, Botnar Research Centre, Oxford, OX3 7LD, UK. [32]Centre for Diabetes and Metabolic Medicine, Barts and The London, Royal London Hospital, Whitechapel, London, E1 1BB, UK. [33]Bone Research Group, Department of Medicine and Therapeutics, University of Aberdeen, Aberdeen, AB25 2ZD, UK. [34]Clinical Pharmacology and Barts and The London Genome Centre, William Harvey Research Institute, Barts and The London School of Medicine and Dentistry, Queen Mary University of London, Charterhouse Square, London EC1M 6BQ, UK. [35]Institute of Cellular Medicine, Musculoskeletal Research Group, 4th Floor, Catherine Cookson Building, The Medical School, Framlington Place, Newcastle upon Tyne, NE2 4HH, UK. [36]Gastroenterology Unit, Radcliffe Infirmary, University of Oxford, Oxford, OX2 6HE, UK. [37]Centre National de Genotypage, 2, Rue Gaston Cremieux, Evry, Paris 91057, France. [38]Department of Gastroenterology & Hepatology, University of Newcastle upon Tyne, Royal Victoria Infirmary, Newcastle upon Tyne NE1 4LP, UK. [39]ALSPAC Laboratory, Department of Social Medicine, University of Bristol, BS8 2BN, UK. [40]Division of Nutritional Sciences, King's College London School of Biomedical and Health Sciences, London SE1 9NH, UK. [41]NIHR-Leeds Musculoskeletal Biomedical Research Unit, University of Leeds, Chapel Allerton Hospital, Leeds, West Yorkshire LS7 4SA, UK. [42]Department of General Internal Medicine, Ninewells Hospital and Medical School, Ninewells Avenue, Dundee DD1 9SY, UK. [43]Department of Clinical Neurosciences, University of Cambridge, Addenbrooke's Hospital, Hills Road, Cambridge, CB2 2QQ, UK. [44]Clinical and Academic Rheumatology, Kings College Hosptal National Health Service Foundation Trust, Denmark Hill, London SE5 9RS, UK. [45]University of Toronto, St. Michael's Hospital, 30 Bond Street, Toronto, Ontario M5B 1W8, Canada. [46]University of Bath, Claverdon, Norwood House, Room 5.11a Bath Somerset BA2 7AY, UK. [47]Division of Community Health Sciences, St George's, University of London, London SW17 0RE, UK. [48]Departments of Health Sciences and Genetics, University of Leicester, 217 Adrian Building, University Road, Leicester, LE1 7RH, UK. [49]Diabetes Research Group, School of Clinical Medical Sciences, Newcastle University, Framlington Place, Newcastle upon Tyne NE2 4HH, UK. [50]Medicine and Therapeutics, Aberdeen Royal Infirmary, Foresterhill, Aberdeen, Grampian AB9 2ZB, UK. [51]School of Medicine and Biomedical Sciences, University of Sheffield, Sheffield, S10 2JF, UK. [52]Nuffield Department of Orthopaedics, Rheumatology and Musculoskeletal Sciences, Nuffield Orthopaedic Centre, University of Oxford, Windmill Road, Headington, Oxford, OX3 7LD, UK. [53]UBC Institute of Mental Health, 430-5950 University Boulevard Vancouver, British Columbia, V6T 1Z3, Canada. [54]Diamantina Institute of Cancer, Immunology and Metabolic Medicine, Princess Alexandra Hospital, University of Queensland, Ipswich Road, Woolloongabba, Brisbane, Queensland, 4102, Australia. [55]Cardiovascular Medicine, University of Oxford, Wellcome Trust Centre for Human Genetics, Roosevelt Drive, Oxford OX3 7BN, UK. [56]Genetics of Diabetes, Peninsula College of Medicine and Dentistry, University of Exeter, Barrack Road, Exeter, EX2 5DW, UK. [57]Clinical and Molecular Genetics Unit, Institute of Child Health, University College London, 30 Guilford Street, London WC1N 1EH, UK. [58]The Wellcome Trust, Gibbs Building, 215 Euston Road, London NW1 2BE, UK. [59]Leicester NIHR Biomedical Research

Unit in Cardiovascular Disease, Glenfield Hospital, Leicester, LE3 9QP, UK. [60]Oxford NIHR Biomedical Research Centre, Churchill Hospital, Oxford, OX3 7LJ, UK. [61]Telethon Institute for Child Health Research, Centre for Child Health Research, University of Western Australia, 100 Roberts Road, Subiaco, Western Australia 6008. [62]Cambridge Institute for Medical Research, University of Cambridge School of Clinical Medicine, Cambridge CB2 0XY, UK. [63]Department of Psychosis Studies, NIHR Biomedical Research Centre for Mental Health at the Institute of Psychiatry, King's College London and The South London and Maudsley NHS Foundation Trust, Denmark Hill, London SE5 8AF, UK. [64]Department Epidemiology and Population Health, London School of Hygiene and Tropical Medicine, London WC1E 7HT and Dept Epidemiology and Public Health, University College London WC1E 6BT, UK. [65]Neuropsychiatric Genetics Research Group, Institute of Molecular Medicine, Trinity College Dublin, Dublin 2, Eire. [66]Department of Oncology, Old Road Campus, University of Oxford, Oxford OX3 7DQ, UK, Digestive Diseases Centre, Leicester Royal Infirmary, Leicester LE7 7HH, UK and Centre for Digestive Diseases, Queen Mary University of London, London E1 2AD, UK. [67]Clinical Neurosciences, St George's University of London, London SW17 0RE, UK.[68]Biomedical Research Centre, Ninewells Hospital and Medical School, Dundee DD1 9SY, UK.[69]NIHR Biomedical Research Centre for Ophthalmology, Moorfields Eye Hospital NHS Foundation Trust and UCL Institute of Ophthalmology, London EC1V 2PD, UK. [70]Department Molecular Neuroscience, Institute of Neurology, Queen Square, London WC1N 3BG, UK.
